# ﻿Two new species of *Rhizoplaca* (Lecanoraceae) from Southwest China

**DOI:** 10.3897/mycokeys.101.115678

**Published:** 2024-01-25

**Authors:** Yanyun Zhang, Yujiao Yin, Lun Wang, Christian Printzen, Lisong Wang, Xinyu Wang

**Affiliations:** 1 College of Life Sciences, Anhui Normal University, 241000, Wuhu, China; 2 Yunnan Key Laboratory for Fungal Diversity and Green Development, Kunming Institute of Botany, Chinese Academy of Sciences, 650201, Kunming, China; 3 Senckenberg Research Institute and Natural History Museum, 60325, Frankfurt am Main, Germany; 4 Key Laboratory for Plant Diversity and Biogeography of East Asia, Kunming Institute of Botany, Chinese Academy of Sciences, 650201, Kunming, China

**Keywords:** new taxa, *Rhizoplacachrysoleuca*-complex, *R.melanophthalma*-complex, saxicolous lichen

## Abstract

In this study, two new species, *Rhizoplacaadpressa* Y. Y. Zhang & Li S. Wang and *R.auriculata* Y. Y. Zhang, Li S. Wang & Printzen, are described from Southwest China, based on their morphology, phylogeny and chemistry. In phylogeny, the two new species are monophyletic, and sister to each other within *Rhizoplacachrysoleuca*-complex. *Rhizoplacaadpressa* is characterized by its placodioid and closely adnate thallus, pale green and heavily pruinose upper surface, narrow (ca. 1 mm) and white free margin on the lower surface of marginal squamules, the absence of a lower cortex, and its basally non-constricted apothecia with orange discs that turn reddish-brown at maturity. *Rhizoplacaauriculata* is characterized by its squamulose to placodioid thallus, yellowish green and marginally pruinose squamules, wide (1−3 mm) and bluish-black free margin on the lower surface of marginal squamules, the absence of a lower cortex, and its basally constricted apothecia with persistently orange discs. *Rhizoplacaadpressa* and *R.auriculata* share the same secondary metabolites of usnic and placodiolic acids.

## ﻿Introduction

*Rhizoplaca* was established by Zopf (1905), solely to accommodate the type species, *R.opaca* (Ach.) Zopf. This species has since been synonymized to *R.melanophthalma* (Ram.) Leuckert et Poelt according to the priorities established by Nomenclature Codes (Leuckert et al. 1977). The genus *Rhizoplaca* was delimited as possessing an umbilicate thallus, with a distinct upper cortex, rather loose medulla, and thick lower cortex (Arup and Grube 2000; Leuckert et al. 1977). However, one umbilicate species, *R.peltata* (DC.) Leuckert & Poelt, was transferred to *Protoparmeliopsis* M. Chiosy, and several placodioid species, including *Lecanoraopiniconensis* Brodo, *L.phadrophthalma* Poelt, *L.novomexicana* H. Magn. were included in *Rhizoplaca* based on molecular phylogenetic results (Zhao et al. 2016). Therefore, the genus circumscription of *Rhizoplaca* requires further investigation.

To date, the genus *Rhizoplaca* includes ca. 25 species that have a worldwide distribution, with the exception of Australia, for which records are lacking (Leuckert et al. 1977; Leavitt et al. 2013a; Zhao et al. 2016; Zhang et al. 2020; Brinker et al. 2022). Recent studies uncovered extensive cryptic species diversity among the cosmopolitan species of *Rhizoplaca*, including *R.chrysoleuca* (Sm.) Zopf, *R.melanophthalma*, *R.phaedrophthalma* and *R.subdiscrepans* (Nyl.) R. Sant (Zhou et al. 2006; Leavitt et al. 2011, 2013a, 2016; Szczepańska et al. 2020). Five new species were described in the *R.melanophthalma*-complex, based on molecular phylogenetic results (Leavitt et al. 2013b). However, the species delimitation of the *R.chrysoleuca*-complex, *R.phaedrophthalma*-complex and *R.subdiscrepans*-complex remains largely unresolved. Our previous study on the genus *Squamarina* verified that the type species of S.sectionPetroplaca Poelt, *Squamarinacallichroa* (Zahlbr.) Poelt (Poelt 1958), belongs to *Rhizoplacachrysoleuca*-complex, on the basis of their orange apothecial disc, *Lecanora*-type ascus and the phylogenetic evidence (Zhang et al. 2020). After our extensive field investigations, many similar specimens were collected in Southwest China. A detailed morphological, phylogenetic and chemical study of these materials proved that they are distinct from *R.callichroa* (Zahlbr.) Y. Y. Zhang and represented two species new to science.

## ﻿Materials and methods

### ﻿Morphological and chemical analyses

Seventy-one specimens from the *Rhizoplacachrysoleuca*-complex and related species were examined in this study. All the specimens were deposited in the Lichen Herbarium of Kunming Institute of Botany (KUN-L) unless stated otherwise. A dissecting microscope, Nikon SMZ745T, was used to observe the morphological features. Apothecia and thalli were sectioned by hand with a razor blade and their microscopic traits were observed and measured using a Nikon Eclipse Ci-S microscope. The macro- and micro- photographs were taken by Nikon digital camera head DS-Fi2, and Nikon D850 camera, respectively. Lugol’s iodine (I) was used to examine the apical structure of asci and 10% potassium hydroxide (KOH) (K) to test whether the granules in the apothecia and thalli dissolved. Lactophenol cotton blue (LCB) was used to dye the hyphae in the microscopic study. Saturated aqueous solution of sodium hypochlorite (NaClO) (C) and 1,4-Phenylenediamine in ethanol solution (P) were applied for spot tests. We sampled ca. 1 mm^2^ apex of the thallus of each dry or fresh specimen for the purpose of thin layer chromatography (TLC) analysis using the solvent systems of A, B and C (Orange et al. 2001).

### ﻿DNA extraction, amplification and sequencing

We took a ca. 1 mm^2^ fragment of the thallus apex from each fresh or dry specimen to extract genomic DNA, following the instructions of the AxyPrep Multisource Genomic DNA Miniprep Kit 50-prep (Qiagen). Polymerase chain reactions (PCR) were performed in an automatic thermocycler (C 1000TM). Five markers, nrITS, nrLSU, RPB1, RPB2 and mtSSU, were chosen for our phylogenetic studies using the primers of ITS1f (Gardes and Bruns 1993) and ITS4a (Larena et al. 1999), LR0R (Rehner and Samuels 1994) and LR5 (Vilgalys and Hester 1990), gRPB1a (Stiller and Hall 1997) and fRPB1c (Matheny et al. 2002), RPB2-6f and RPB2-7cr (Liu et al. 1999), mrSSU1 mrSSU3R (Zoller et al. 1999), respectively. Amplifications were performed with a total volume of 25 μl, containing 12.5 μl 2× MasterMix [TaqDNA Polymerase (0.1 units/μl), 0.4 mM MgCl_2_, 0.4 nM dNTPs] (Aidlab Biotechnologies Co. Ltd.), 0.5 μl of each primer, 10 μl ddH_2_O and 1 μl of DNA. The PCR settings per locus are provided in Table 1. PCR products were sequenced by TsingKe Biological Technology using the same primers which had been used for amplification (Kunming, China).

**Table 1.** The PCR settings used for each marker.

Program	nrITS & nrLSU	RPB1 & RPB2	mtSSU
Initial denaturation	95 °C 5 min	94 °C 5 min	94 °C 5 min
Phase 1	10 cycles	34 cycles	4 cycles
	95 °C 30 s	94 °C 45 s	94 °C 30 s
	66 °C 30 s	52 °C 50 s	54 °C 30 s
	72 °C 1 min 30 s	72 °C 1 min	72 °C 1 min
Phase 2	34 cycles		30 cycles
	95 °C 30 s		94 °C 30 s
	56 °C 30 s		50 °C 30 s
	72 °C 1 min 30 s		72 °C 1 min
Final extension	72 °C 10 min	72 °C 10 min	72 °C 10 min

### ﻿Phylogenetic analyses

The raw sequences were initially checked with the BLAST tool on the NCBI online service (https://blast.ncbi.nlm.nih.gov/Blast.cgi) to make sure that they belonged to lichenized fungi. According to previous studies, we selected two species of the genus *Protoparmeliopsis* and two species of *Polyozosia* A. Massal. as the outgroup for the genus *Rhizoplaca* (Medeiros et al. 2021; Zhao et al. 2016; Zhang et al. 2020). Geneious R8 was used to assemble the raw sequences and generate one matrix per locus. The matrices were individually aligned with MAFFT using the web service (https://mafft.cbrc.jp/alignment/server/index.html) (Katoh et al. 2019; Kuraku et al. 2013). For alignment, we used the G-INS-1 strategy and default parameters, with the exception of the offset value, which was set as 0.2. Because of the possible incongruence between nuclear genes and mitochondrial genes, we concatenated only the nrITS, nrLSU, RPB1 and RPB2 regions as a 4-loci dataset using the program SequenceMatrix v. 1.7.8 to reconstruct the phylogenetic tree of *Rhizoplaca*. PartitionFinder 2 (Lanfear et al. 2017) was used to estimate the best schemes and nucleotide substitution models for maximum likelihood (ML) and Bayesian inference (BI) analyses. The best schemes and selected models are shown in Table 2.

**Table 2.** The best schemes and nucleotide substitution models selected by PartitionFinder, based on the 4-loci dataset.

Partition scheme	Model
Subset1 (nrITS1, nrITS2)	GTR+G
Subset2 (5.8S)	K80+I
Subset3 (nrLSU)	TRNEF+I
Subset4 (RPB1-B codon1, RPB1-C codon1, RPB2-7 codon1)	TRN+G
Subset5 (RPB1-C codon2, RPB1-B codon2, RPB2-7 codon2)	F81+I
Subset6 (intron of RPB1, RPB1-B codon3, RPB1-C codon3, RPB2-7 codon3)	K80+G

Bayesian reconstruction of phylogeny based on the 4-loci dataset was performed with MrBayes v. 3.1.2 (Huelsenbeck and Ronquist 2001), using four Markov chains running for one hundred million generations with two runs. Trees were sampled every 1000 generations. The first 25% of runs were discarded as burn-in. Subset rates were modelled as fixed and equal. We used the default distributions for priors. We considered the sampling of the posterior distribution to be adequate when the average standard deviation of split frequencies was < 0.01. Tracer v. 1.6 (Rambaut and Drummond 2003) was used to assess the chain convergence by checking the effective sampling size (ESS > 200). ML analyses were performed with RaxmlHPC, using the General Time Reversible model of nucleotide substitution (GTR). Support values were inferred from the 70% majority-rule tree of all saved trees obtained from 1000 non-parametric bootstrap replicates. Trees were visualized in Mega 7 and edited in PowerPoint.

## ﻿Results and discussion

**Figure 1.**
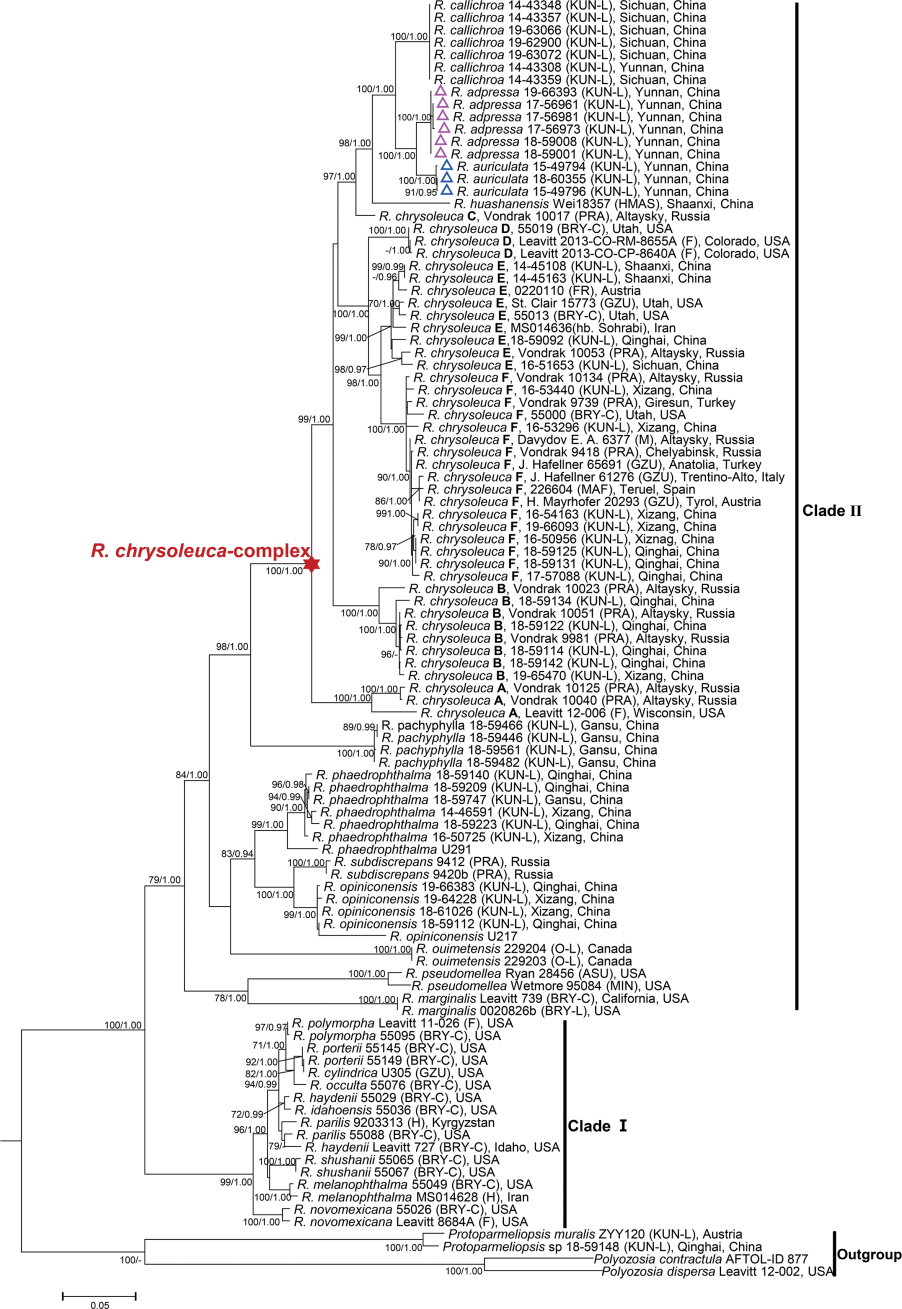
Maximum Likelihood tree for the genus *Rhizoplaca*, based on a 4-loci (nrITS, nrLSU, RPB1 and RPB2) concatenated dataset. Maximum Likelihood bootstrap values ≥ 70 and posterior probabilities ≥ 0.90 are displayed on adjacent branches. The two new species are marked by triangles.

153 new sequences from eight species of the genera *Rhizoplaca* and *Protoparmeliopsis* were obtained in this study (Table 3). Phylogenetic trees were reconstructed based on a 4-loci dataset including 103 samples of 26 species (Fig. 1). Our results were in accordance with the results of previous studies that species of *Rhizoplaca* are split into two main clades (Zhao et al. 2016; Szczepańska et al. 2020; Zhang et al. 2020; Brinker et al. 2022). Clade I (ML = 99; BI = 1.00) included a placodioid species, *Rhizoplacanovomexicana*, two vagrant species, *R.idahoensis* and *R.haydenii*, and the *R.melanophthalma*-complex. The species delimitation of *R.melanophthalma*-complex are largely dependent on the molecular data (Leavitt et al. 2013b). Species in Clade I are characterized by the bluish-black, rarely yellowish discs and mainly distributed in North America (Ryan and Nash 1991; Leavitt et al. 2011). Clade II (ML = 79; BI = 1.00) consisted of *R.chrysoleuca*-complex, *R.subdiscrepans*-complex, *R.phaedrophthalma*-complex and several other species lineages, including *R.pachyphylla*, *R.marginalis*, *R.pseudomellea* and *R.ouimetensis*.

**Table 3.** Sequences used in this study; newly obtained sequences are shown in boldface.

Species	Locality*	Voucher specimens	Accession number*				
			nrITS	nrLSU	RPB1	RPB2	mtSSU
*Polyozosiacontractula*	NA	AFTOL-ID 877	HQ650604	DQ986746	DQ986817	DQ992428	DQ986898
*P.dispersa*	USA	Leavitt 12-002	KT453733	NA	KT453888	KT453921	NA
*Protoparmeliopsismuralis*	Austria: Salzburg	ZYY120 (KUN-L)	**OR669100**	**OR669126**	**OR712769**	**OR712777**	**OR681862**
*Protoparmeliopsis* sp.	China: Qinghai	18-59148 (KUN-L)	**OR669101**	**OR669127**	**OR712770**	**OR712778**	**OR681863**
*Rhizoplacaadpressa*	China: Yunnan	17-56961 (KUN-L)	**OR669102**	NA	NA	**OR712779**	NA
*R.adpressa*	China: Yunnan	17-56981 (KUN-L)	**OR669103**	**OR669128**	NA	**OR712780**	NA
	China: Yunnan	17-56973 (KUN-L)	**OR669104**	**OR669129**	NA	**OR712781**	NA
	China: Yunnan	19-66393 (KUN-L)	**OR669105**	NA	NA	**OR712782**	NA
	China: Yunnan	18-59008 (KUN-L)	**OR669106**	NA	NA	NA	NA
	China: Yunnan	18-59001 (KUN-L)	**OR669107**	NA	NA	NA	NA
*R.auriculata*	China: Yunnan	18-60355 (KUN-L)	**OR669108**	**OR669130**	**OR712771**	**OR712783**	NA
	China: Yunnan	15-49794 (KUN-L)	**OR669109**	**OR669131**	**OR712772**	**OR712784**	NA
	China: Yunnan	15-49796 (KUN-L)	**OR669110**	**OR669132**	**OR712773**	**OR712785**	NA
*R.callichroa*	China: Sichuan	14-43348 (KUN-L)	MK778045	NA	NA	NA	NA
	China: Sichuan	14-43357 (KUN-L)	MK778046	NA	NA	NA	NA
	China: Sichuan	14-43359 (KUN-L)	MK778043	NA	NA	NA	NA
	China: Yunnan	14-43308 (KUN-L)	MK778044	NA	NA	NA	NA
	China: Sichuan	19-63066 (KUN-L)	**OR669111**	NA	NA	NA	NA
	China: Sichuan	19-63072 (KUN-L)	**OR669112**	NA	NA	NA	NA
	China: Sichuan	19-62900 (KUN-L)	**OR669113**	NA	NA	NA	NA
*R.chrysoleuca* ‘A’	USA: Wisconsin	Leavitt 12-006 (F)	KU934562	NA	NA	KU935053	NA
	Russia: Altaysky	Vondrak 10125 (PRA)	KU934565	NA	KU935314	KU935056	NA
	Russia: Altaysky	Vondrak 10040 (PRA)	KU934567	NA	KU935316	KU935058	NA
*R.chrysoleuca* ‘B’	China: Qinghai	18-59134 (KUN-L)	**OR995297**	**OR995320**	**PP049801**	**PP054345**	**PP001783**
	China: Qinghai	18-59122 (KUN-L)	**OR995298**	**OR995321**	**PP049802**	**PP054346**	**PP001784**
	China: Qinghai	18-59114 (KUN-L)	**OR995299**	**OR995322**	**PP049803**	**PP054347**	**PP001785**
	China: Qinghai	18-59142 (KUN-L)	**OR995300**	**OR995323**	**PP049804**	**PP054348**	**PP001786**
	China: Xizang	19-65470 (KUN-L)	**OR995301**	**OR995324**	NA	NA	**PP001787**
	Russia: Altaysky	Vondrak 9981 (PRA)	KU934568	NA	KU935317	KU935059	NA
	Russia: Altaysky	Vondrak 10023 (PRA)	KU934570	NA	NA	KU935061	NA
	Russia: Altaysky	Vondrak 10051 (PRA)	KU934571	NA	NA	KU935062	NA
*R.chrysoleuca* ‘C’	Russia: Altaysky	Vondrak 10017 (PRA)	KU934573	NA	KU935318	KU935064	NA
*R.chrysoleuca* ‘D’	USA: Utah	55019 (BRY-C)	HM577254	NA	KU935319	KU935065	NA
	USA: Colorado	Leavitt 2013-CO-CP-8640A (F)	KU934575	NA	KU935320	KU935067	NA
	USA: Colorado	Leavitt 2013-CO-RM-8655A (F)	KU934577	NA	KU935321	KU935069	NA
*R.chrysoleuca* ‘E’	USA: Utah	55013 (BRY-C)	HM577248	NA	KU935325	KU935073	NA
	Iran: East Azarb aijan	MS014636 (hb. Sohrabi)	KT453731	NA	KU935322	KU935070	NA
	Russia: Altaysky	Vondrak 10053 (PRA)	KU934582	NA	KU935330	KU935078	NA
	China: Shaanxi	14-45108 (KUN-L)	**OR995302**	**OR995325**	NA	NA	NA
	China: Shaanxi	14-45163 (KUN-L)	**OR995303**	**OR995326**	NA	NA	**PP001788**
	Austria	0220110 (FR)	**OR995304**	NA	NA	NA	NA
	USA: Utah	St. Clair 15773 (GZU)	**OR995305**	NA	NA	NA	NA
	China: Qinghai	18-59092 (KUN-L)	**OR995306**	**OR995327**	**PP049805**	**PP054349**	**PP001789**
	China: Sichuan	16-51653 (KUN-L)	**OR995307**	**OR995328**	**PP049806**	NA	NA
*R.chrysoleuca* ‘F’	China: Xizang	16-53440 (KUN-L)	**OR995308**	**OR995329**	NA	**PP054350**	**PP001790**
	China: Xizang	16-53296 (KUN-L)	**OR995309**	**OR995330**	**PP049807**	**PP054351**	NA
	Russia: Altaysky	Davydov E. A. 6377 (M)	**OR995310**	NA	NA	NA	NA
	Turkey: Anatolia	Hafellner J. 65691 (GZU)	**OR995311**	NA	NA	NA	NA
	Italy: Trentino-Alto	Hafellner J. 61276 (GZU)	**OR995312**	NA	NA	NA	NA
	Austria: Tyrol	Mayrhofer H. 20293 (GZU)	**OR995313**	NA	NA	NA	NA
	China: Xizang	16-54163 (KUN-L)	**OR995314**	**OR995331**	NA	**PP054352**	**PP001791**
	China: Xizang	19-66093 (KUN-L)	**OR995315**	**OR995332**	**PP049808**	NA	NA
	China: Xizang	16-50956 (KUN-L)	**OR995316**	**OR995333**	NA	**PP054353**	**PP001792**
	China: Qinghai	18-59125 (KUN-L)	**OR995317**	**OR995334**	NA	**PP054354**	NA
	China: Qinghai	18-59131 (KUN-L)	**OR995318**	**OR995335**	**PP049809**	**PP054355**	**PP001793**
	China: Qinghai	17-57088 (KUN-L)	**OR995319**	**OR995336**	**PP049810**	**PP054356**	**PP001794**
	USA: Utah	55000 (BRY-C)	HM577233	NA	KU935335	KU935084	NA
	Russia: Chelyabinsk	Vondrak 9418 (PRA)	KU934593	NA	KU935344	KU935093	NA
	Spain: Teruel	226604 (MAF)	KU934596	NA	NA	NA	NA
	Turkey: Giresun	Vondrak 9739 (PRA)	KU934597	NA	KU935347	KU935096	NA
	Russia: Altaysky	Vondrak 10134 (PRA)	KU934608	NA	KU935349	KU935098	NA
*R.cylindrica*	USA	U305 (GZU)	AF159941	NA	NA	NA	NA
*R.haydenii*	USA	55029 (BRY-C)	HM577298	NA	KU935352	KU935102	NA
	USA: Idaho	Leavitt 727 (BRY-C)	NA		KT453902	KT453932	NA
*R.huashanensis*	China: Shaanxi	Wei18357 (HMAS-L)	AY530885	NA	NA	NA	NA
*R.idahoensis*	USA	55036 (BRY-C)	HM577297	NA	KU935367	KU935116	NA
*R.marginalis*	USA: California	Leavitt 739 (BRY-C)	KT453732	NA	KT453901	KT453936	NA
	USA	0020826b (BRY-L)	KU934655	NA	KU935370	KU935123	NA
*R.melanophthalma*	USA	55049 (BRY-C)	HM577270	NA	JX948324	JX948362	NA
	Iran	MS014628 (H)	JX948271	NA	JX948317	JX948355	NA
*R.novomexicana*	USA	55026 (BRY-C)	HM577257	NA	KU935390	KU935136	NA
	USA	Leavitt 8684A (F)	KU934708	NA	KU935391	KU935137	NA
*R.occulta*	USA	55076 (BRY-C)	HM577307	NA	JX948344	JX948383	NA
*R.opiniconensis*	NA	U217	AF159928	NA	NA	NA	NA
	China: Xizang	19-64228 (KUN-L)	**OR669116**	**OR669135**	NA	NA	NA
	China: Qinghai	19-66383 (KUN-L)	**OR669117**	**OR669136**	NA	NA	NA
	China: Xizang	18-61026 (KUN-L)	**OR669118**	NA	NA	NA	NA
	China: Qinghai	18-59112 (KUN-L)	**OR669119**	**OR669137**	**OR712775**	**OR712788**	**OR681865**
*R.ouimetensis*	Canada	229203 (O-L)	ON943161	NA	NA	NA	NA
	Canada	229204 (O-L)	ON943160	NA	NA	NA	NA
*R.pachyphylla*	China: Gansu	18-59466 (KUN-L)	MK778048	NA	MK766417	MK766436	MN192152
	China: Gansu	18-59446 (KUN-L)	MK778047	NA	MK766416	MK766435	MN192151
	China: Gansu	18-59482 (KUN-L)	MK778049	NA	MK766418	MK766437	MN192153
	China: Gansu	18-59561 (KUN-L)	MK778050	NA	MK766419	MK766438	MN192154
*R.parilis*	Kyrgyzstan	9203313 (H)	JX948193	NA	KU935392	KU935138	NA
	USA	55088 (BRY-C)	HM577319	NA	JX948313	JX948352	NA
*R.phaedrophthalma*	NA	U291	AF159938	NA	NA	NA	NA
	China: Xizang	14-46591 (KUN-L)	**OR669120**	**OR669138**	NA	NA	**OR681866**
	China: Qinghai	18-59223 (KUN-L)	**OR669121**	**OR669139**	NA	**OR712789**	**OR681867**
	China: Qinghai	18-59140 (KUN-L)	**OR669122**	**OR669140**	NA	**OR712790**	**OR681868**
*R.phaedrophthalma*	China: Qinghai	18-59209 (KUN-L)	**OR669123**	**OR669141**	NA	**OR712791**	**OR681869**
	China: Gansu	18-59747 (KUN-L)	**OR669124**	**OR669142**	NA	**OR712792**	**OR681870**
	China: Xizang	16-50725 (KUN-L)	**OR669125**	**OR669143**	**OR712776**	**OR712793**	**OR681871**
*R.polymorpha*	USA	55095 (BRY-C)	HM577326	NA	KU935411	KU935159	NA
	USA	Leavitt 11-026 (F)	JX948194	NA	JX948328	JX948366	NA
*R.porterii*	USA	55149 (BRY-C)	HM577380	NA	JX948341	JX948380	NA
	USA	55145 (BRY-C)	HM577376	NA	JX948340	JX948379	NA
*R.pseudomellea*	USA	Wetmore 95084 (MIN)	MN931737	NA	NA	NA	NA
	USA	Ryan 28456 (ASU)	MN931733	NA	NA	NA	NA
*R.shushanii*	USA	55065 (BRY-C)	HM577286	NA	JX948334	JX948372	NA
	USA	55067 (BRY-C)	HM577288	NA	JX948335	JX948373	NA
*R.subdiscrepans*	Russia	9412 (PRA)	KU934899	NA	NA	NA	NA
	Russia	9420b (PRA)	KU934901	NA	NA	NA	NA

*NA = not available.

The two new species, *Rhizoplacaadpressa* (ML = 100; BI = 1.00) and *R.auriculata* (ML = 100; BI = 1.00), formed highly supported monophyletic clade, and were grouped together as sister clades within the *R.chrysoleuca*-complex. The large genetic variation within the *R.chrysoleuca*-complex has been shown in multiple previous studies (Cansaran et al. 2006; Zhou et al. 2006; Zheng et al. 2007). Leavitt et al. (2016) delimited six species-level clades within this complex, provisionally called *Rhizoplacachrysoleuca* ‘A’, ‘B’, ‘C’, ‘D’, ‘E’ and ‘F’. Our phylogenetic trees showed that *R.chrysoleuca* ‘B’, ‘E’ and ‘F’ were also present in China. To some extent, these clades are morphologically different. Thallus of *R.chrysoleuca* ‘B’ is placodioid, whereas *R.chrysoleuca* ‘E’ and *R.chrysoleuca* ‘F’ are umbilicate that usually contain a conspicuous umbilicus on the lower surface. *R.chrysoleuca* ‘E’ differs from *R.chrysoleuca* ‘F’ in its yellowish thalline margins. However, the species delimitation of *R.chrysoleuca* s. str. and above clades still needs future studies, including the check of type specimen, secondary metabolites and the detailed morphological features. The species, *R.callichroa*, *R.huashanensis*, together with the two new species, *R.adpressa* and *R.auriculata*, formed a monophyletic clade that forms a sister group to *R.chrysoleuca* ‘C’. However, these species differ from *R.chrysoleuca* by their broadly ellipsoid to subfusiformis ascospores (Wei 1984; Zhang et al. 2020). *Rhizoplacahuashanensis* is the basal species of this clade and differs in its black apothecial disc, the presence of a lower cortex, and its restricted distribution in Northwest China (Wei 1984). *Rhizoplacacallichroa* formed a sister clade to *R.adpressa* and *R.auriculata* but was distinguished by the pale brown lower surface (Zhang et al. 2020).

To date, ten species of Clade II in *Rhizoplaca* have been reported from China: *R.adpressa*, *R.auriculata*, *R.callichroa*, *R.chrysoleuca* (representing multiple lineages), *R.fumida*, *R.huashanensis*, *R.pachyphylla*, *R.subdiscrepans*, *R.opiniconensis* and *R.phaedrophthalma* (Gao 1987; Zhao et al. 2016; Lü et al. 2020; Wei 2020; Zhang et al. 2020). The species *R.fumida* has been synonymized to *R.chrysoleuca* based on morphological and phylogenetic analyses (Wei and Wei 2005). According to a revised circumscription of *R.subdiscrepans* s. str. (Szczepańska et al. 2020), the records of this species in China need more investigation. We provided a key to only the eight species of *Rhizoplaca* Clade II which have been confirmed as present in China. This key should effectively distinguish between these species.

## ﻿Taxonomy

### ﻿ Rhizoplaca adpressa

Taxon classificationFungiLecanoralesLecanoraceae

Y. Y. Zhang & Li S. Wang sp. nov.

7E900CC4-82A4-5ACB-8E08-97B4531A19A3

851059

Fig. 2

#### Type.

China. Yunnan Prov.: Kunming Ci., Shilin Co., 24°41′N, 103°22′E, 1883 m, on calcareous rock, 25 October 2017, Li S. Wang et al. 17-56973 (KUN-L0066051).

#### Diagnosis.

The species *Rhizoplacaadpressa* is characterized by its placodioid and closely adnate thallus, pale green and heavily pruinose upper surface, lower surface of marginal squamules with a white and narrow free margin, the absence of lower cortex, and the basally non-constricted apothecia with orange disc that turn reddish-brown at maturity.

#### Etymology.

The epithet refers to the thallus, which is closely adnate to the substratum.

#### Description.

Thallus placodioid, umbilicate at least when young, rosulate, 1−3.5 cm across, centrally areolate, areoles continuous, plane, ca. 0.5 mm in diam., marginally squamulose, squamules radiating, 1−2.5 mm across. Upper surface pale green, heavily pruinose, smooth, rarely cracked, matt, lower surface with a white and narrow (ca. 1 mm) free margin, without tomentum. Upper cortex 13−20 μm thick, filled with pale brown (soluble in K) and brown (insoluble in K) granules, consisting of thin-walled and short-celled hyphae, 1.5−2.5 μm in diam., length of cell 3−7 μm, epinecral 10−16 μm thick, filled with brown granules, partly soluble in K, algal layer continuous, filled with black substance, insoluble in K, 67−75 μm thick, algae 8.5−12 μm in diam., medulla filled with black substance, insoluble in K, lower cortex lacking.

Apothecia common, laminal, scattered to slightly grouped, lecanorine, originally at same level with thallus, without thalline margin, then adnate, not constricted at base, 0.5−1 mm in diam. Apothecial disc orange, reddish-brown with age, pruinose, plane to slightly convex, thalline margin entire, thinner than 0.1 mm, concolorous with thallus. Hymenium filled with orangish and gray granules, insoluble in K, 58−70 μm high, epihymenium non-gelatinized, filled with brown (soluble in K) and orange granules (insoluble in K), weakly interspersed, 12−16 μm thick, parathecium extremely reduced, subhymenium with orangish gray granules, insoluble in K, 12.5−20 μm, hypothecium colorless, with orange and brown granules, insoluble in K, 50−180 μm, algae under hypothecium not continuous, irregularly grouped, cortex of thalline margin same as upper cortex, even, ca. 25 μm thick, paraphyses simple, ca. 3 μm in diam., septate, length of cell 10−13 μm, asci clavate, 50−55 × 15−22 μm, ascospores broadly ellipsoid to subfusiformis, hyaline, 9.5−13 × 6.5−9 μm. Pycnidia rare, conidia filiform, 16−25 × ca. 0.7 μm.

#### Chemistry.

K+ pale yellow, C-, P-; usnic and placodiolic acids were detected in TLC.

#### Distribution and ecology.

The new species only grows on exposed hard calcareous rock in karst landform at elevations of 1883−2623 m in Yunnan Province, China.

#### Notes.

*Rhizoplacacallichroa* is similar to this new species but differs in its yellowish green upper surface, the apothecia constricted at base when mature, and the persistently orange apothecial disc (Zhang et al. 2020). *Rhizoplacahuashanensis* is similar to *R.adpressa* but differs in its black lower surface that contains a lower cortex, and its restricted distribution in Shaanxi (Northwest China) (Wei 1984). *Rhizoplacachrysoleuca* differs from *R.adpressa* in its larger apothecia (0.5−6 mm in diam.) and marginal lobes (2−5 mm long, 1−3 mm wide), a wide and bluish-black free margin on lower surface, the presence of gelatinized lower cortex, and the persistently orange apothecia with constricted base. *Rhizoplacaphaedrophthalma* also has reddish-brown apothecial disc when mature, but differs in the lobate thallus with yellowish and epruinose upper surface, the strongly convex disc, and the smaller ascospores, 7–10 × 4.5–7 μm (Lü et al. 2020; Poelt 1958).

**Figure 2.**
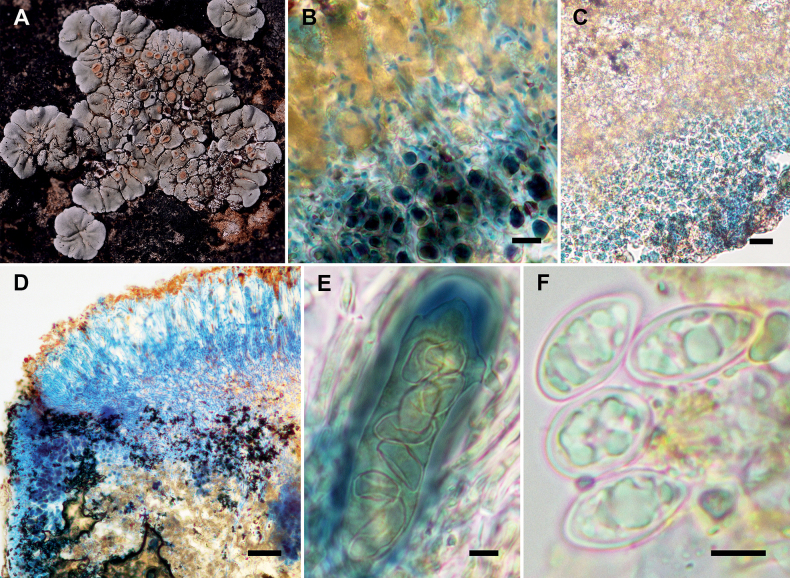
*Rhizoplacaadpressa* (KUN-L0066051) **A** holotype **B** hyphae of upper cortex (LCB) **C** lower surface lacks lower cortex (LCB) **D** section of apothecia (LCB) **E** ascus (Lugol’s solution) **F** ascospores (water). Scale bars: 10 μm (**B**); 20 μm (**C**); 50 μm(**D**); 5 μm (**E, F**).

#### Additional specimens examined.

China. Yunnan Prov.: Dali, Heqing Co., Songgui Town, 26°18′N, 100°10′E, 2229 m, on calcareous rock, 20 June 2018, Li S. Wang et al. 18-58987 (KUN-L0065133), 18-58988 (KUN-L0065134), 18-59991 (KUN-L0065137), 18-58997 (KUN-L0065143), 18-59001 (KUN-L0065147), 18-59008 (KUN-L0065154), 18-59935 (KUN-L0063742), 18-59937 (KUN-L0063744), 18-59940 (KUN-L0063747), same location, 26°18′N, 100°10′E, 2260 m, on calcareous rock, 29 August 2005, Li S. Wang, D. L. Niu & H. Luo 05-25135 (KUN-L0040473); Kunming Ci., Shilin Co., 24°41′N, 103°22′E, 1883 m, on calcareous rock, 25 October 2017, Li S. Wang et al. 17-56961 (KUN-L0066046), 17-56965 (KUN-L0062405), 17-67966 (KUN-L0062443), 17-56981 (KUN-L0076202), 17-57054 (KUN-L0062534), same location, 24°42′N, 103°21′E, 1890 m, on calcareous rock, 19 September 2003, Li S. Wang 03-22617 (KUN-L0040472), same location, 1910 m, on calcareous rock, 11 May 2008, Li S. Wang 08-29555 (KUN-L0040474), same location, 1900 m, on calcareous rock, 19 February 2010, Li S. Wang 10-31345 (KUN-L0048845); Lijiang Ci., Ning lang Co., Yongning Vil., 27°43′N, 100°40′E, 2675 m, on calcareous rock, 27 July 2020, Li S. Wang et al. 20-66488 (KUN-L0076274); Yulong Co., Mt. Yulong, 26°56′N, 100°12′E, 2623 m, on calcareous rock, 31 December 2019, Li S. Wang & Y. Y. Zhang 19-66393 (KUN-L0076201).

### ﻿ Rhizoplaca auriculata

Taxon classificationFungiLecanoralesLecanoraceae

Y. Y. Zhang, Li S. Wang & Printzen sp. nov.

9A7FF03C-F356-569B-840B-A008BB69D125

851060

Fig. 3

#### Type.

China. Yunnan Prov.: Deqin Co., Benzilan Vil., besides Jinsha River, 28°11′N, 99°21′E, 2099 m, on chloritoid schist, 19 August 2018, Li S. Wang et al. 18-60139 (KUN-L0065413).

#### Diagnosis.

The species is characterized by the yellowish green upper surface, ear-like marginal squamules containing a bluish-black and wide, free lower margin, the lack of lower cortex, and the persistently orange apothecia with constricted base.

#### Etymology.

The epithet refers to the ear-like margins of marginal squamules.

#### Description.

Thallus squamulose to placodioid, umbilicate at least when young, rosulate or not, 2−5 cm across, centrally squamulose, squamules continuous to irregularly overlapped, slightly convex, 1−2.5 mm across, marginal squamules radiating or not, larger than the center, 2−4 mm across, with ear-like margins. Upper surface yellowish green, epruinose to only pruinose at margins of squamules, smooth to rugose, lower surface with a bluish-black free margin, 1−3 mm wide, no tomentum. Upper cortex 16−22 μm thick, filled with pale brown granules, soluble in K, upper part with scattered brown granules, insoluble in K, consisting of thin-walled and short-celled hyphae, 2−3 μm in diam., length of cell 3−7 μm, epinecral 10−25 μm thick, filled with brown granules, soluble in K, algal layer continuous, 67−80 μm thick, filled with black substance, insoluble in K, algae 8.5−12 μm in diam., medulla filled with black substance, insoluble in K, lower cortex lacking.

**Figure 3.**
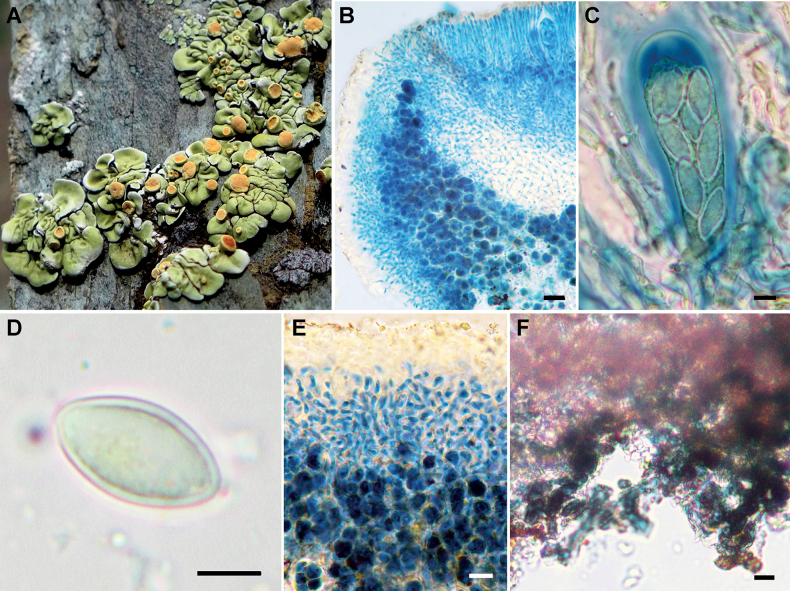
*Rhizoplacaauriculata* (KUN-L0065413) **A** holotype **B** section of apothecia (K and LCB) **C** asci and ascospores (Lugol’s solution) **D** ascospore (water) **E** upper cortex and epinecral (K and LCB) **F** lower surface with bluish-black hyphae lacks lower cortex (LCB). Scale bars: 20 μm (**B**); 5 μm (**C, D**); 10 μm (**E, F**).

Apothecia common, laminal, scattered to slightly grouped, lecanorine, sessile, constricted at base, 0.5–2 (3) mm in diam., disc orange, pruinose, plane to slightly convex, thalline margin entire, 0.1–0.2 mm wide, concolorous with thallus, pruinose. Hymenium filled with orange and gray granules, insoluble in K, 75−87 μm high, epihymenium non-gelatinized, filled with brown (soluble in K) and orange granules (insoluble in K), not interspersed, 12.5−19 μm thick, parathecium extremely reduced, subhymenium with gray granules, insoluble in K, 17−30 μm, hypothecium colorless, with grouped brown granules, insoluble in K, 60−100 μm, algae under hypothecium continuous to irregularly grouped, cortex of thalline margin same as upper cortex, even, 25−30 μm thick, paraphyses simple to slightly branched, ca. 3 μm in diam., septate, length of cell 9−14 μm, tips slightly thickened, asci clavate, 62−75 × 15−21 μm, ascospores broadly ellipsoid to subfusiformis, hyaline, 10−16 × 6.5−9.5 μm. Pycnidia immersed in the thallus, ostioles not seen, conidia filiform, straight to curved, 22.5−37.5 × 0.7 μm.

#### Chemistry.

K+ pale yellow, C-, P-; usnic and placodiolic acids detected in TLC.

#### Distribution and ecology.

The new species only grows on dry and exposed calcareous chloritoid schist at elevation of 2000–2108 m beside the Jinsha River in Sichuan and Yunnan Provinces, China.

#### Notes.

*Rhizoplacacallichroa* is similar to this new species in thallus and apothecia size but differs by its pale brown, lower free margins (Zhang et al. 2020) and the substratum of hard calcareous rock in karst landform. *R.huashanensis* shares yellowish green upper surface and black lower surface with *R.auriculata*, but differs in the presence of a lower cortex, black apothecial discs, smaller ascospores (11.55−12.32 × 6.93−7.70 μm), and the absence of placodiolic acid (Wei 1984). *R.chrysoleuca* differs from *R.auriculata* in its thallus with gelatinized lower cortex and the smaller ascospores (7.5−11.5 × 4−5.8 μm). *R.adpressa* differs from *R.auriculata* in its thallus with areolate center and squamulose margins, pale green upper surface with white heavy pruina, the lower surface with white free margins, and the adnate apothecia with orange to reddish-brown discs.

#### Additional specimens examined.

China. Sichuan Prov.: Derong Co., Benzilan Vi., besides Jinsha River, 28°12′N, 99°20′E, 1960 m, on chloritoid schist, 4 October 2009, Li S. Wang & J. Wang 09-31121 (KUN-L0048841). Yunnan Prov.: Deqin Co., Benzilan Vi., besides Jinsha River, 28°11′N, 99°21′E, 2099 m, on chloritoid schist, 19 August 2018, Li S. Wang et al. 18-60136 (KUN-L0065415), 18-60336 (KUN-L0065496), same location, 2108 m, on chloritoid schist, 19 August 2018, Li S. Wang et al. 18-60352 (KUN-L0065512), 18-60355 (KUN-L0065515), same location, 28°23′N, 99°01′E, 2000 m, on chloritoid schist, 31 October 2015, Li S. Wang, Y. Y. Zhang & M. X. Yang 15-49794 (KUN-L0040537), 15-49796 (KUN-L0040538), same location, 28°10′N, 99°23′E, 2115 m, on chloritoid schist, 27 August 2006, Li S. Wang, Oh Soon-OK & D. L. Niu 06-26670 (KUN-L0040471), 06-26684 (KUN-L0040575), same location, 28°10′N, 99°31′E, 2110 m, on rock, 27 August 2006, H. Harada 23764 (KUN-L0051510).

### ﻿Key to the species of *Rhizoplacachrysoleuca*-complex and related species in China

**Table:** 

1	lower cortex absent	**2**
–	lower cortex present	**5**
2	apothecial disc black	***R.pachyphylla***
–	apothecial disc orange to reddish-brown	**3**
3	lower surface contains bluish-black free margin	***R.auriculata***
–	lower surface contains white or pale brown free margin	**4**
4	thallus closely adnate to the substratum, centrally areolate, areoles ca. 0.5 mm in diam., apothecia adnate, not constricted at base, apothecial disc orange when young, reddish-brown when mature	***R.adpressa***
–	thallus relatively loosely adnate to the substratum, centrally squamulose, squamules 1−2 mm in diam., apothecia constricted at base when mature, apothecial disc persistently orange	***R.callichroa***
5	thallus umbilicate, apothecial disc pruinose	**6**
–	thallus placodioid, apothecial disc epruinose	**7**
6	apothecial disc orange	***R.chrysoleuca* (representing multiple lineages)**
–	apothecial disc black	***R.huashanensis***
7	apothecial disc reddish-brown, upper surface completely yellowish-green	***R.phaedrophthalma***
–	apothecial disc yellowish-brown, upper surface yellowish-green with marginal lobes having an orange pigmented apex	***R.opiniconensis***

## Supplementary Material

XML Treatment for Rhizoplaca adpressa

XML Treatment for Rhizoplaca auriculata

## ﻿Additional information

### Author ORCIDs

Yanyun Zhang https://orcid.org/0000-0002-0902-5066

Christian Printzen https://orcid.org/0000-0002-0871-0803

Xinyu Wang https://orcid.org/0000-0003-2166-6111

### Data availability

All of the data that support the findings of this study are available in the main text.
